# Association between poor sleep and mental health issues in Indigenous communities across the globe: a systematic review

**DOI:** 10.1093/sleepadvances/zpae028

**Published:** 2024-05-02

**Authors:** Dan Richard Fernandez, Rennie Lee, Nam Tran, Dure Sameen Jabran, Stephanie King, Lisa McDaid

**Affiliations:** Institute for Social Science Research, University of Queensland, Indooroopilly QLD, Australia; Institute for Social Science Research, University of Queensland, Indooroopilly QLD, Australia; Tobacco, Alcohol and Other Drugs Unit, Australian Institute of Health and Welfare, Bruce, ACT, Australia; Frazier Institute, Translational Research Institute, University of Queensland, Woolloongabba, QLD, Australia; Centre for Rural and Remote Health Murtupuni Campus, James Cook University, Mount Isa, QLD, Australia; Institute for Social Science Research, University of Queensland, Indooroopilly QLD, Australia

## Abstract

**Study Objectives:**

Evidence from studies among non-Indigenous populations has established the association of poor sleep to mental health issues and supported how improving sleep could reduce the risk of mental ill health. In contrast, for Indigenous people, who experience disproportionate rates of mental ill health, the association between sleep and mental health and the potential of sleep health in reducing the risk and severity of mental health issues have never been fully reviewed. Considering the literature gap, this review assesses the association between sleep and mental health in Indigenous people.

**Methods:**

Following PRISMA guidelines, a study was submitted to the PROSPERO database for registration (293798) prior to commencing the review. Then academic databases were searched for relevant studies published up till 19 February 2023. Studies with quantitative data on sleep and mental health association in Indigenous people were included and a narrative review/synthesis was conducted.

**Results:**

Seven studies, using carer/self-reports (six cross-sectional, one longitudinal) among three Indigenous groups (*N* = 3066) met the inclusion criteria. In Indigenous Australian children, arousal problems were associated with aggression, and withdrawn behavior, while early bedtime was associated with a lower risk of behavioral problems. In Native American young people, insomnia symptoms were associated with depressive symptoms in adults, short sleep was associated with affective disorders. Clinical sleep issues, i.e. restless leg and apnea, were associated with depression. In Amerindian/Mestizo adults, restless leg syndrome was associated with depression and anxiety. Overall, findings report the prevalence of poor sleep and mental health issues among Indigenous communities across the globe. Six studies scored “moderate quality” and one study scored “high quality” in quality assessment.

**Conclusions:**

While there is limited research available, our finding suggests an association between poor sleep and mental health issues in Indigenous people. Further investigation of the potential role of, and investing in, sleep health could help support mental health.

Indigenous people, the custodians of the oldest continuing cultures on the planet, descended from and identified with the original inhabitants of a given region before it was conquered by colonial societies [1, 2]. Worldwide, over 470 million people identify as Indigenous people (6% of the global population), living in 70 countries from the Arctic to the South Pacific [1–3]. Indigenous people have strong connections to their land, community, and culture, which are inextricably linked to their identities, and physical and spiritual well-being [1, 3].

For centuries in Australia, Indigenous people lived uninterrupted until European colonization began [4]. Colonization resulted in violence (including genocide and massacres), the introduction of new diseases (e.g. smallpox, measles, influenza, and venereal diseases), and the dispossession of their ancestral lands [5–7]. Assimilation policies were also instituted based on the belief in white superiority, which sought for social, cultural, and spiritual practices to be erased [7, 8]. Assimilation was instituted by forcibly removing Indigenous children from their families and coercing them to adopt a white culture which included not speaking in their native languages and not using the names given by their parents [8]. Decades of unexpressed grief and anger have transferred through generations and have resulted in intergenerational trauma [9]. The impact of intergenerational trauma and historical as well as ongoing inequities, racism, and discrimination manifest in the form of adverse physical health outcomes, poor mental health and well-being, reduced quality of life, and lower life expectancy than their counterparts [6, 9–11].

The impact of intergenerational trauma as reflected by the state of Indigenous people’s mental health is concerning [12]. A global overview of suicide rates in countries with colonial histories, such as Australia, Aotearoa/New Zealand, Canada, and the United States report significantly higher rates of mental health issues and death caused by suicide among Indigenous people compared to their counterparts [13–19].

While there are no available overall statistics on the current worldwide trends of mental health and death caused by suicide among Indigenous people, the country data reports are concerning. In Australia, statistics from the Australian Institute of Health and Welfare (AIHW) 2018–2019 highlights that one in four Indigenous Australians people reported mental health or behavioral conditions, with anxiety as the most reported mental health condition (17%), followed by depression (13%). Psychological distress is also identified as a concerning issue affecting one in three Indigenous Australian adults, particularly those living in non-remote areas [20]. The mental health issue in Indigenous Australian communities is also a key contributor to high rates of deaths due to self-harm in Indigenous communities [15]. Among Indigenous Australians aged 5 to 17 years, suicide was over five times the rate for non-Indigenous young people between 2010 and 2014 [21]. In 2021, figures from the Closing the Gap campaign report that the suicide age-standardized rate for Aboriginal and Torres Strait Islander people increased to 27.1 per 100 000 people (for New South Wales, Queensland, Western Australia, South Australia and the Northern Territory combined) from 25.1 per 100 000 people in 2018 [22]. Similarly, the Māori, Indigenous people of New Zealand, are disproportionately affected by mental health issues like anxiety, depression, and mental distress compared to non-Māori [16]. A study by Sullivan et al. (2017) reported up to 71% of the participants aged 18–34 years reported problems with anxiety/depression [17]. Consequently in 2018, despite the Government’s initiatives, Maori still have the highest suicide rates, at 21.7 per 100 000, in contrast to 14.7 per 100 000 for non-Maori [23]. In the United States of America, the Center for Disease Control reports that American Indian/Native American people experience serious psychological distress 2.5 times more than the general population over a month’s time [24]. And deaths caused by suicide among American Indian/Native American people between the ages of 15–19 is more than double that of non-Hispanic whites [24]. In Canada, Indigenous people aged 15 years or older were less likely to report positive mental health compared to their non-Indigenous counterparts [18]. Furthermore, the Survey of Safety in Public and Private Spaces (2018) reported that less than half (45.8%) of the First Nations population reported excellent or very good mental health while almost two-thirds (62.3%) of non-Indigenous people reported the same [19]. Meanwhile, in terms of deaths caused by suicide Webster (2016) compares Canadian Government statistics and a study commissioned by the Inuit people (Indigenous people of Canada). Government statistics claim that suicide rates in the four Inuit regions are more than six times higher than the rate in non-Indigenous regions [25]. The Canadian Government statistics report, among Inuit youth, suicide is responsible for 40% of deaths, compared with only 8% in the rest of Canada [25]. In contrast, the Inuit-commissioned study states that Inuit suicide rate is 11 times the Canadian average—or 55% higher than the Canadian Government report [25].

Considering these statistics, Indigenous communities, mental health service providers, researchers, and policymakers are working together to identify solutions that are culturally appropriate, effective, and sustainable. For example, designing a holistic and culturally embedded mental health system, promoting cultural relevant protective factors (e.g. traditional upbringings and maintaining culture, and family and social support), culturally appropriate service delivery, and the integration of traditional and biomedical knowledge [12, 26–28].

In understanding the mental health issues affecting Indigenous people, it is imperative to consider every available strategy to address them. In this review, mental health issues refer to “mental health conditions.” The World Health Organization’s WHO explains that “mental health conditions” is the broader term used to describe “mental disorders, psychosocial disabilities and (other) mental states associated with significant distress, impairment in functioning, or risk of self-harm” [29].

To address mental health issues/conditions, an under-recognized public health strategy is Sleep health [30]. Sleep health promotion impacts a wide range of health outcomes, including mental health [30]. In fact, studies from non-Indigenous populations have established a strong link between poor sleep and mental health issues [31–33], and established that improvement in sleep health can lead to improved mental health [34–36]. Unfortunately, the contribution of poor sleep to mental health issues in Indigenous people has not been fully reviewed [34, 36, 37]. Among the reasons is the paucity of studies on sleep-mental health among Indigenous. To the best of our knowledge, there is only one systematic review report on the association between poor sleep and mental health issues among Indigenous people in North America [38]. It suggested that poor sleep is associated with an increased risk of mental distress, depression, and anxiety [38].

Aside from the limited literature, another challenge in studying the association between poor sleep and mental health issues among Indigenous people may be the differing perspectives on mental health. Essentially, for Western, non-Indigenous people, mental health is focused on “how individuals think and believe, and how they adapt to and partake in regular day-to-day existence” [39]. While it may involve “associations with companions, close family, and outsiders,” the focus is essentially on the individual [39]. In contrast, for Indigenous people, mental health is but a facet of an encompassing construct known as “social and emotional wellbeing” (SEWB). National Strategic Framework for Aboriginal and Torres Strait Islander peoples’ Mental Health and Social and Emotional Well-being 2017–2023 explains: “In broad terms, social and emotional well-being is the foundation for physical and mental health for Aboriginal and Torres Strait Islander peoples. It is a holistic concept which results from a network of relationships between individuals, family, kin, and community. It also recognizes the importance of connection to land, culture, spirituality and ancestry, and how these affect the individual” [40]. SEWB includes the “social, emotional, and cultural well-being of the whole community throughout the entire life-course” [40]. This comprehensive perspective includes society-level concepts such as social justice, equity, and rights, as well as traditional knowledge, traditional healing, and connection to country [41, 42] and “encompass[es] mental health and physical, cultural, and spiritual health” [42].

Furthermore, the same differing perspectives are observed in sleep health. Fatima et al. (2021) observed that Indigenous Australians’ conceptualization of sleep health was different from the Western interpretation of sleep health. Fatima et al. (2021) observe that an important component of sleep health among Indigenous people which is “the connection between dreams and sleep is not adequately captured in current (Western/mainstream) tools and resources to promote sleep health” (p. A33) [43].

Another important but largely unexplored aspect of sleep health is how sleep loss which inevitably results in dream loss (due to shortened rapid eye movement REM sleep) affects Indigenous people [44]. In general, according to research, while reduced REM/dreaming—including REM sleep and dream recall—is closely associated with depression, appropriate REM/dreams facilitate healthy emotional processing [44, 45]. However, for Indigenous people, REM/dream loss has even deeper implications because their SEWB is inextricably linked to culture [46]. Indigenous communities in different parts of the world have documented how creativity and knowledge in their cultures have been shaped by revelation through dreams [46]. For example, among Indigenous Australians, an account of artist Roy Bagay Wiggan, a Bardi Elder who creates objects of art or *Ilma*, totems used in ceremonial dance and ritual [47], recounts how *Ilma* is revealed to him by deceased relations in dreams [46]. Similarly, Aubrey Tigan, a respected elder and lawman of the Bardi and Djawi peoples, shared recounts of an old man in his dreams who would keep coming and telling him to carve that shell [48]. Hence for Indigenous peoples, the effect of dream loss may impact culture which in turn affects SEWB.

Considering the disproportionately high rates of mental health issues in Indigenous communities and the deeper implication of sleep health to Indigenous culture, it is important to review the evidence on the role of sleep in the mental health of Indigenous people to guide future research and inform strategies for integrating sleep in mental health programs and services. To accomplish this, all available studies assessing the association of sleep (both quantity and quality) and mental health issues of Indigenous people which were measured both subjectively and objectively were considered.

In recognizing the lack and the gap in the literature, this review aims to (1) assess the state of the literature on sleep and mental connection in Indigenous communities, (2) explore the strength and direction of association between poor sleep and mental health outcomes, and (3) highlight key gaps in the literature to offer recommendations for future research.

## Materials and Methods

### Systematic review protocol

We finalized the PRISMA (Preferred Reporting Items for Systematic Reviews and Meta-Analyses) checklist and protocol which were then submitted for registration to the PROSPERO database (293 798) in December 2021. Literature searches were commenced thereafter.

### Search strategy and selection criteria

In our online and manual search, we included all published studies if the study explored the role of poor sleep in mental health issues, focused on Indigenous people, and was published in the English language. Studies were excluded if the association between poor sleep and mental health was explored in a group of people with underlying medical conditions, pregnant women, or shift workers; the study explored sleep and mental health issues for Indigenous and non-Indigenous peoples but did not provide separate data for Indigenous people; or the article was published as clinical guidelines, opinion piece or letter to the editor. First author (DRF) searched key academic databases, e.g. CINAHL; Cochrane; Elsevier/Science Digest; ProQuest; PsycINFO; PubMed; SCOPUS; Google Scholar, and the web page of the Indigenous Health InfoNet, from December 2021 to February 2022 and updated in February 2023. In addition, conference papers, conference poster abstracts, and reports were also considered. The search strategy included controlled vocabulary terms and keywords, e.g. “poor sleep”; “sleep disorder”; “inadequate sleep”; “anxiety”; “mental health issues”; “psychological issues”; “Indigenous”; “First Nations Peoples.” While there are multiple ways to define “poor sleep,” for the purpose of this review, we have defined poor sleep as problems in any dimension of sleep, i.e. quality, timing, duration, efficiency, and sleepiness during waking hours interfering with the refreshing and restorative nature of sleep [49]. Issues in mental health among Indigenous peoples were identified through a non-validated self-report [50] and scores from carer/self-reported validated instruments such as the Child Behavior Checklist (CBL) [51], and Depression Anxiety Stress Scales–21 (DAS-21) [52]. Considering the diversity of Indigenous peoples across the globe, it is difficult to have an all-encompassing definition that captures the rich and unique cultural values, beliefs, and practices of different Indigenous people groups. Nonetheless, to define the scope of this work, in this review, we followed the United Nations’ interpretation of the term “Indigenous peoples” as the ethnic group who descended from and identified with the original inhabitants of a given region [53]. The published identified studies that met inclusion criteria were collated.

### Data extraction and quality assessment

**Figure 1.**
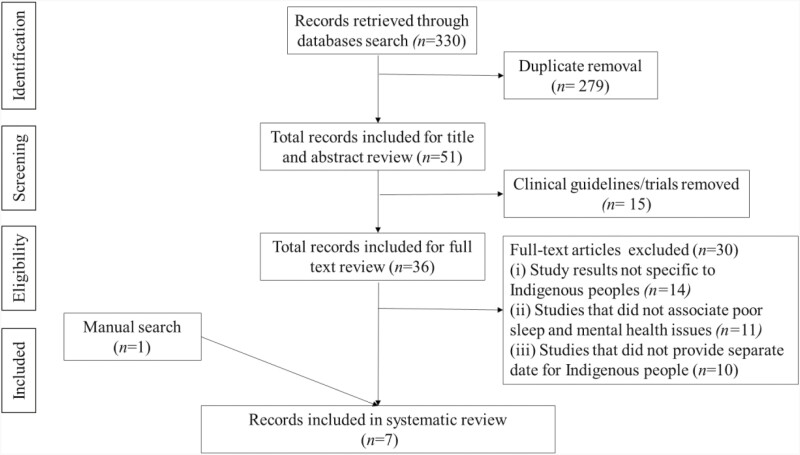
Screening of literature on the association between poor sleep and mental health issues among Indigenous people.

The importing and first screening of titles was conducted by the first author (DRF) using the management software Covidence [54]. After removing the duplicate articles, two reviewers (DRF and DSJ) conducted the second screening by independently reviewing the studies. Studies meeting the inclusion criteria were selected. Disagreements in study inclusion/exclusion were resolved through consensus. Next, the following key data from the selected studies were extracted by the first author (DRF): (1) general information (author’s name, publication year), (2) study aims, (3) study and participants’ characteristics (design, sample size, demographics), (4) data collection methods/tools (5) findings, (6) limitations, and (7) strengths. A Preferred Reporting Items for Systematic Reviews and Meta-Analyses (PRISMA) flowchart diagram shows the number of articles retrieved, screened, excluded, and selected (Figure 1).

This review is guided by strengths-based approaches recognizing the cultural strength of Indigenous people, including connection to country and culture, spirituality, ancestral ties, resilience, kinship, community leadership, and governance [55]. This review is also guided by the expertise and experience of an Indigenous Australian coauthor and cultural mentor (SK) to ensure that the interpretation and reporting of study findings are culturally respectful and responsive to Indigenous peoples’ historical and contemporary circumstances. The methodological quality (risk of bias) of the studies was assessed using the National Institutes of Health (NIH): Quality Assessment Tool for Observational Cohort and Cross-Sectional Studies [56]. The NIH tool comprises 14 items assessing the selection and non-response bias (external validity), measurement bias, and analysis bias (internal validity). Based on the quality assessment scores, studies were grouped as “high quality” (low risk of bias), “moderate quality” (moderate risk of bias), and “poor quality” (high risk of bias). Two reviewers (DRF and DSJ) independently assessed the risk of bias. Each study was assessed for Indigenous leadership and involvement in the research process. Hence, the level of involvement, participation in community benefits, and adherence to local cultural protocols were highlighted. For this, the adapted version of the Aboriginal and Torres Strait Islander Quality Appraisal Tool was used [57]. This tool comprises 14 items assessing the adherence with ethical and methodological standards specific Indigenous research.

Based on the scores, Indigenous leadership and involvement in the research was categorized as “low,” “moderate,” and “high.” Two reviewers (DRF and DSJ) independently assessed Indigenous leadership and involvement. When a consensus was not reached, Indigenous coauthor and cultural mentor (SK) arbitrated.

### Data analysis

Considering the heterogeneity in research design of the studies, we utilized narrative review/synthesis for our data analysis. The narrative review/synthesis is a qualitative approach wherein the findings of other studies are combined without using statistical methods [58, 59].

## Results

Of 330 screened research articles, seven studies; one longitudinal study [60], and six cross-sectional studies [50–52, 61–63] published between 2008 and 2021, with a total population of 3075 [1295 children (ages 6.3–11), 321 adolescents (ages 11.1–18), young adults (ages 18.1–26), and, 1459 adults (ages 31.35–59.5)] met the inclusion criteria. All the studies were community-based and utilized purposive sampling. Three studies were from the United States of America [50, 62, 63], two from Australia [36, 51], one from Canada [61], and one from Ecuador [52]. Two studies focused on children [36, 51], two studies on adolescents/youth and young adults [50, 62], and three studies focused on adults [52, 61, 63] (Table 1).

**Table 1.** Characteristics of Included Studies Covering Poor Sleep and Mental Health Issues of Indigenous Communities Across the Globe [50–52, 60–63]

Author (year)	Study aim and location	Participant demographics	Study design	Study variables	Key findings	Strengths and limitation/s
Froese et al. (2008) [61]	Assess the prevalence of sleep symptoms and the relationships between sleep-related symptoms and depression among Native American/ American Indians from three First Nations North American groups (Gitxsan, Nisga’a, and Tsimshian) in British Columbia, Canada.	438 adults (56% females, mean age 43.2 years, SD ± 14.3)	Cross-sectional	*Independent variable/s:* self-reported sleepiness measured utilizing the Epworth Sleepiness Scale (ESS); symptoms of Obstructive Sleep Apnea and restless leg syndrome *Outcome:* self-reported depression measured using the Personal Health Questionnaire (PHQ-9) *Other variables:* age and sex, anthropometric data (neck circumference and BMI) and self-reported medical history (smoking habit, alcohol use, use of anti-depressant medication, use of prescription sleep medication, use of herbal, health food, traditional sleep remedies; diabetes, hypertension, and heart attack)	The risk for depression was significantly correlated in participants reporting restless leg syndrome (OR: 1.82; 95% CI: 0.53 to 3.12,), insomnia symptoms (OR: 4.49; 95% CI: 3.14 to 5.83), and apnea (OR: 2.46; 95% CI: 0.47 to 4.46)	*Strengths:* validated measures for data collection *Limitations:* Cross-sectional design
Blunden et al. (2010) [51]	Assess the association between sleep problems and emotional and behavioral problems in 50 First Nations and non-Indigenous children from Darwin in the Northern Territory and Palmerston in Australia	25 children (56% males, mean age: 8.8 years, SD ± 1.4)	Cross-sectional	*Independent variable:* parent-reported sleep problems; arousal problems, sleep–wake transition problems, excessive daytime sleepiness, hyperhidrosis, and total sleep problems assessed using the Sleep Disturbance Scale for Children *Dependent variable/s:* parent-reported behavior issues and school performance assessed using a validated measure Child Behavior Checklist (CBL) *Covariates:* parent-reported age, sex, and parental education	Arousal problems were positively correlated with externalized behaviors (*r* = 0.32, *p*-value: .02), specifically aggression (*r* = 0.37, *p*-value:.009), withdrawn behavior (*r* = 0.31, *p*-value:.02) and total behaviors (*r* = 0.43, *p*-value:.001)	*Strengths:* validated measures for data collection *Limitations:* small sample, low participation rate (30.6%), parent/carer report, cross-sectional design
Arnold et al. (2013) [62]	Examine the impact of sleep and other factors on depressive symptoms and suicidality among American Indian adolescents from the Lumbee tribe in Robeson or a neighboring county North Carolina, USA.	80 youth (59.5% females, mean age 13.7, SD ± 13.7)	Cross-sectional	*Independent variable/s:* self-reported sleepiness measured through the Epworth Sleepiness Scale (ESS) and average time in bed (TIB) per night. *Outcome:* Depressive symptoms were measured using the Center of Epidemiologic Studies Depression Scale for Children (CES-DC) *Covariates:* age, sex, grade in school, and sexual orientation, weight and body mass index, mental illness, self-esteem, and cultural connectedness	Multivariable regression result suggests that time in bed is not linked with depressive symptoms (*β*: − 0.501, *p*-value:.71), but significantly reduced the odds of suicidal ideation (OR:0.62, *p*-value:04)	*Strengths:* validated measures for data collection Limitations: Cross-sectional design
Farrell (2013) [50]	Examine the relationship between sleep disturbances and suicidality in students American Indians/Native Americans students from 132 schools in the United States	232 adolescent and young adults (57% females mean age, 15.34 years, SD ± 1.81)	Cross-sectional	*Independent variable/s:* self-reported insomnia symptoms, i.e. trouble falling asleep or staying asleep. *Dependent variable/s:* self-reported depressive symptoms, suicidal ideation, and suicide attempts *Covariates:* age and sex	Insomnia symptoms were significantly associated with depression (OR: 4.87, 95% CI: 2.4 to 9.89), but did not significantly predict suicidal ideation (OR: 1.96, 95% CI: 0.96 to 4.02) or suicide attempts (OR: 1.25, 95% CI: 0.38 to 4.14)	*Limitations:* cross-sectional design, non-validated tools for data collection
Castillo et al. (2014) [52]	Assess the association between restless leg syndrome and mental health issues in 665 First Nations people from Atahualpa in rural coastal Ecuador in South America	665 adults (42% males, mean age 59.5 years SD ± 12.6)	Cross-sectional	*Independent variable:* self-reported restless legs syndrome assessed using validated measure International Restless Legs Syndrome Study Group (IRLSSG) field instrument Dependent variable/s: self-reported depression anxiety and stress assessed using Depression Anxiety Stress Scales–21 Covariates: age and sex	Restless leg syndrome was associated with significantly higher odds of depression (OR: 4.5, 95% CI: 2.2 to 9.7;), anxiety (OR: 3.6, 95% CI: 1.7 to 7.7), and stress (OR: 3.3 95%CI: 1.5-7.6)	*Strengths:* validated measures for data collection *Limitations:* Cross-sectional design
Ehlers et al. (2017) **[63]**	Assess interaction between sleep and anxiety, and affective disorders in American Indian community sample from eight contiguous rural Indian reservations in the United States	356 adults (54% females; mean age 31.35 years SD ± 14.4)	Cross-sectional	*Independent variable:* self-reported sleep quality measured utilizing the Pittsburgh Sleep Quality Index (PSQI) *Dependent variable:* self-reported major affective and anxiety disorders, measured utilizing the Semi-Structured Assessment for the Genetics of Alcoholism (SSAGA-II) *Covariates:* age, sex, education, civil status, household income, occupation, American Indian ancestry, cultural identification, physical and medical data (body mass index, current drinking frequency, current drinking quantity, self-reported diabetes, use of sleep medication, nicotine dependence, alcohol use disorder, cannabis use disorder, and stimulant use disorder)`	Participants who had short sleep (< 6 h) had a significantly higher experience of anxiety (16%) and affective disorders (16%) than their counterparts.	*Strengths:* validated measure for sleep quality assessment *Limitations:* cross-sectional design
Fatima et al. (2021) [60]	Assess the role of sleep trajectories (4½ to 6 years) in emotional and behavioral problems (9½ to 11 years) in 1270 Aboriginal and Torres Strait Islander children in Australia	1270 children (49.4% males, mean age: 6.3 years, SD ± 1.5)	Longitudinal wave 5 to wave 10 of the Footprints in Time cohort (2015-2019)	*Independent variable:* sleep trajectories derived from parent-reported sleep duration, weekday bedtimes, wake-time, and sleep problems *Dependent variable/s:* parent-reported emotional and behavioral problems (assessed using Strengths and Difficulties Questionnaire) *Covariates:* Parent-reported age, sex and family size, structure, and composition; and cultural attachment.	Children in the Early sleepers//early riser trajectory had lower odds of being in the high emotional and behavioral problem trajectory group. (OR: 0.48, 95% CI: 0.28 to 0.82)	*Strengths:* Longitudinal design large population study, data collected across 11 remotes communities in Australia, validated measure for outcome assessment *Limitations:* parent-reported data, non-validated measure for assessing sleep issues

### Poor sleep and mental health issues

All the studies used either parent/carer or self-reports to record sleep and mental issues. To assess sleep, two studies utilized Epworth Sleepiness Scale [61, 62], one study utilized the Pittsburgh Sleep Quality Index [63], one study utilized the International Restless Legs Syndrome Study Group field instrument [52], one study utilized the Sleep Disturbance Scale for Children [51], one study utilized parent-reported child’s sleep patterns and issues (duration, weekday bedtimes, wake-time, and sleep problems) [60], and one study used non-validated single-item based measures to assess sleep issues [50]. To assess the prevalence of mental health issues, the majority of studies used validated measures such as the Strengths and Difficulties Questionnaire [60], the Child Behavior Checklist (CBL) [51], Semi-Structured Assessment for the Genetics of Alcoholism (SSAGA-II) [63] Personal Health Questionnaire (PHQ-9) [61], Depression Anxiety Stress Scales–21 (DAS-21) [52], Center of Epidemiologic Studies Depression Scale for Children (CES-DC) [64].

### Short sleep duration

There was variation in the definition of short sleep in the studies included in the review. While for adolescents, short sleep was conceptualized as sleeping for less than 8 hours, for adults, short sleep was defined as sleeping for less than 6 hours [63]. Short sleep duration was reported in the sample populations of two studies included in the review. In adolescents, short sleep was reported among 29·3% (*N* = 80) people from the Native American community in North Carolina, USA [62]. However, the prevalence of short sleep in adults was comparatively lower, as only 16% (*N* = 356) of adults from an Native American community from eight contiguous rural Indian reservations in the United States reported sleeping for less than 6 hours per night [63].

### Sleep problems and insomnia symptoms

Sleep problems and insomnia symptoms were reported in the sample populations of three studies in the review. Total sleep problems (arousal problems, sleep–wake transition problems, excessive daytime sleepiness, and hyperhidrosis) were prevalent among 32% (*N* = 25) Indigenous Australian children participants [51]. Insomnia symptoms (trouble falling asleep or staying asleep at least once a week almost every day, or every day in the previous month) were prevalent among 25% (*N* = 232) of adolescent and young adult Native American participants from 132 schools in the United States [50]. While among adults, 17·2% (*N* = 438) of the participants from a Native American group reported insomnia symptoms [61].

### Restless leg syndrome and obstructive sleep apnea

The two clinical sleep issues explored in the sample population of three studies in this review were restless leg syndrome (RLS) and obstructive sleep apnea (OSA). OSA varied from 6·3% (*N* = 438) in adult participants from three Native American groups in Canada [61]. Whereas the prevalence of RLS varied from 6% (*N* = 665) among adults of Amerindian/Mestizo descent participants from Ecuador [52] to 17·7% (*N* = 438) of Native American adults [61].

### Mental health issues

The mental health issues identified in the included studies were behavioral problems, affective disorders, and suicidal ideation and attempts.

### Behavioral problems

Behavioral problems (aggression, withdrawn behavior, and high emotional problems) were reported in two studies covering Indigenous Australian children [60]. The prevalence of behavioral issues varied from 57% (*N* = 25) children from Darwin, Australia and 10·4% (*N* = 1270) children from the Footprints in Time—The Longitudinal Study of Indigenous Children cohort [60].

### Affective disorders

There were five studies reporting the common affective disorders of depression and anxiety in the context of poor sleep. The prevalence of depression in the sample population of Native American adolescents and youth varied from 18% (*N* = 232) [50] to 30.8% (*N* = 80) [62]. In the adult population studied, the prevalence of depression varied from 11% (*N* = 665) in Amerindian/Mestizo adults from Ecuador [61] to 88.8% (*N* = 338) in Native American adults from Canada [61]. There was a prevalence of Diagnostic and Statistical Manual of Mental Health (DSM)-5 disorders among 35·8% (*N* = 356) Native American adults from eight reservations in the United States [63]. The prevalence of anxiety disorders in two adult sample populations varied from 14% (*N* = 665) in Amerindian/Mestizo adults from Ecuador [52] to 23·3% (*N* = 356) Native American adults from the United States [63]

### Suicidal ideation and suicide attempts

In one study among Native American adolescents and young adult sample populations, suicidal ideation was reported at 18% (*N* = 232) 95% CI: NR, while suicide attempts were reported at 6% (*N* = 232; 95% CI: NR) [50].

### Association between poor sleep and mental health issues

In a sample population of Indigenous Australian children, arousal problems were positively correlated with aggression (*r* = 0·37, *p*-value: ·009), withdrawn behavior (*r* = 0·31, *p*-value:·.02) and total behavioral problems (*r* = 0·43, *p*-value:·001) [51]. Farrell et al. reported that in a sample population of Native American adolescents and young adults, insomnia symptoms were significantly associated with depression (OR: 4·87, 95% CI: 2·4 to 9·89) but not suicidal ideation (OR: 1·96, 95% CI: 0·96 to 4·02) or suicide attempts (OR: 1·25, 95% CI: 0·38 to 4·14) [50]. The potentially protective effect of sleep in the young population was highlighted in two studies. One study of Indigenous Australian children found that early bedtime was associated with lower odds of emotional and behavioral problems (OR: 0·48, 95% CI: 0·28 to 0·82) [60]. Another study involving Native American adolescents reported that adequate sleep duration significantly reduced the odds of suicidal ideation (OR: 0·62, p-value:.04) [62].

Among a sample population of Native American adults, short sleep (<6 hours) was significantly associated with anxiety problems [63]. Evidence from Native American groups highlighted that the risk of depression was significantly increased in participants reporting RLS (OR: 1·82; 95% CI: 0·53 to 3·12), insomnia symptoms (OR: 4.49; 95% CI: 3·14 to 5·83) and apnea (OR: 2·46; 95% CI: 0·47 to 4·46) [61]. Likewise, Castillo et al. (2014) reported that among a study population of Amerindian/Mestizo adults from Ecuador, RLS was associated with significantly higher odds of depression (OR: 4·5, 95% CI: 2·2 to 9·7), anxiety (OR: 3·6, 95% CI: 1·7 to 7·7), and stress (OR: 3·3 95% CI: 1·5 to 7·6) [52].

### Study quality and Indigenous leadership and engagement in research

Quality assessment and Indigenous leadership and ownership of research were assessed for each study. The key factors affecting the study quality include non-representative, nonrandom sample, lack of longitudinal data to assess causal links and self-reported self or parent data. All but one study used validated measures for data collection [50]. Also, the studies adjusted for key covariates, e.g. age, gender, socioeconomic status, parental education, cultural identity/connectedness, and health history in the regression model. Six studies rated “moderate quality” (moderate risk of bias) [50–52, 61–63] and one study rated “high quality” (low risk of bias) [60].

Indigenous leadership and involvement in the research process were assessed using the adapted version of the Aboriginal and Torres Strait Islander Quality Appraisal Tool [57]. However, the information provided in the papers was insufficient to effectively evaluate Indigenous leadership and involvement. The first author (DRF) reached out to corresponding authors for further information. The three authors who responded reported the extent of Indigenous leadership and involvement in research as, “low” [63], “moderate” [51], and “high” [60], respectively.

## Discussion

This is the first systematic review to assess the association between poor sleep and mental health issues among Indigenous peoples globally and inform efforts to improve mental health. The findings of this review suggest an association between sleep and mental ill health among Indigenous peoples. However, considering that we found only seven studies from three Indigenous communities in four countries, indicates that the role of poor sleep to mental health among Indigenous people remains under-researched.

In all studies included in this review, high rates of poor sleep (short sleep duration, sleep problems, and insomnia symptoms, RLS, and OSA) were reported among Indigenous groups. This finding is confirmed in a review by Yiallourou et al., (2021) who report that Indigenous people from high-income countries (Australia, Canada, New Zealand, and the United States) have poor sleep quantity and quality [65]. Although the lack of evidence and the diversity of the included Indigenous communities precluded a meta-analysis, the findings of this review are supported by evidence from non-Indigenous communities. For example, meta-analytic evidence, based on longitudinal studies, suggests insomnia as a key predictor of depression and other mental health conditions, e.g. anxiety disorders, bipolar disorder, and suicide [66].

The review does suggest that improving sleep could be an approach used to reduce the risk of mental health issues. Evidence from Indigenous children in Australia suggests that early bedtime is associated with lower odds of emotional and behavioral problems [67] and a cross-sectional study involving Native American adolescents suggests that adequate sleep duration significantly reduced the odds of suicidal ideation [62]. These findings are supported by a meta-analysis of 16 studies of non-Indigenous children and youths from 40 different countries, which reported longer sleep duration was associated with better emotional regulation, and better quality of life/well-being [68].

Understanding the state of sleep health and its association with mental health in both Indigenous people and non-Indigenous people provides insights into strategies to improve sleep. In non-Indigenous populations, to improve sleep health (thereby, improve mental health) systematic reviews have suggested strategies like behavioral/non-pharmacologic sleep programs which include physical activity, relaxation training, environmental modification, stimulus control therapy, sleep restriction therapy, sleep hygiene, cognitive restructuring, and other approaches [69–71]. However, considering the deeper implication of sleep and distinct understanding of SEWB these strategies may not be readily adapted to Indigenous people [40, 44]. Hence, the development of culturally appropriate programs co-designed with Indigenous people may be an important strategy in addressing sleep-mental health [72].

There are limitations to this review. First, there is a lack of information on Indigenous people’s leadership and engagement. While the missing information may be related to constraints such as the lack of reporting guidelines and article word limits, it is recommended that future studies provide detailed information on Indigenous involvement and leadership in research. Second, except for one study [50], all utilized validated sleep and mental health measures; however, these tools were not validated in Indigenous people’s contexts. Since the concept of sleep and SEWB for Indigenous people are distinct from their non-Indigenous counterparts, the data collected in these studies might not fully capture the state of sleep and mental health issues in Indigenous people. Third, six of the seven studies included were cross-sectional; hence, there is no evidence for causality. Fourth, studies in this review included nonrandom purposive sampling, and therefore the findings of this review will have limited generalizability. Fifth, Australian spellings were used for some search terms (e.g. apnoea and behaviour); hence, search results with US spelling of search terms (e.g. apnea and behavior) may have not been included. Finally, this review was also limited to studies published in English, with evidence-based on non-validated sleep assessment measures for Indigenous people.

## Conclusion

The findings of this review suggest an association between poor sleep and mental health issues in Indigenous communities across the globe. This review also confirms the lack of available research literature, which is essential in the development of Indigenous sleep health programs, there is an impetus for more studies on this topic. Finally, to expand on the existing body of knowledge; future research should consider longitudinal evidence from different Indigenous cohorts and explore co-designing culturally appropriate sleep health programs with Indigenous communities.
